# Motion and Flexibility in Human Cytochrome P450 Aromatase

**DOI:** 10.1371/journal.pone.0032565

**Published:** 2012-02-27

**Authors:** Wenhua Jiang, Debashis Ghosh

**Affiliations:** Department of Pharmacology, State University of New York Upstate Medical University, Syracuse, New York, United States of America; Consejo Superior de Investigaciones Cientificas, Spain

## Abstract

The crystal structures of human placental aromatase in complex with the substrate androstenedione and exemestane have revealed an androgen-specific active site and the structural basis for higher order organization. However, X-ray structures do not provide accounts of movements due to short-range fluctuations, ligand binding and protein-protein association. In this work, we conduct normal mode analysis (NMA) revealing the intrinsic fluctuations of aromatase, deduce the internal modes in membrane-free and membrane-integrated monomers as well as the intermolecular modes in oligomers, and propose a quaternary organization for the endoplasmic reticulum (ER) membrane integration. Dynamics of the crystallographic oligomers from NMA is found to be in agreement with the isotropic thermal factors from the X-ray analysis. Calculations of the root mean square fluctuations of the C-alpha atoms from their equilibrium positions confirm that the rigid-core structure of aromatase is intrinsic regardless of the changes in steroid binding interactions, and that aromatase self-association does not deteriorate the rigidity of the catalytic cleft. Furthermore, NMA on membrane-integrated aromatase shows that the internal modes in all likelihood contribute to breathing of the active site access channel. The collective intermolecular hinge bending and twisting modes provide the flexibility in the quaternary association necessary for membrane integration of the aromatase oligomers. Taken together, fluctuations of the active site, the access channel, and the heme-proximal cavity, and a dynamic quaternary organization could all be essential components of the functional aromatase in its role as an ER membrane-embedded steroidogenic enzyme.

## Introduction

Cytochrome P450 aromatase catalyzes the biosynthesis of estrogens from their androgenic precursors by converting the partially unsaturated A-ring to an aromatic A-ring. Structure-function relationships of aromatase have been studied for more than thirty years, but many issues remain unresolved. The recent crystal structure of human placental aromatase showing a compact active site cleft [1] has shed new light on the decades old problems. In the crystal, aromatase molecules are found to form head-to-tail oligomers [2]. This association of monomers is probably driven by electrostatic interactions between the “head” and “tail” segments of two adjacent molecules. Mutagenesis results demonstrate the functional implications of oligomerization of aromatase. Recently, Praporski et al. also reported a high order organization of aromatase in living cells using atomic force microscopy (AFM) and fluorescence resonance energy transfer [3]. The high-resolution AFM images support the formation of aromatase homodimer and oligomers that are stabilized in the lipid bilayer membrane.

However, the dynamical properties of aromatase that may play critical functional roles, such as membrane integration and active site access channel opening, have not yet been addressed. Availability of the crystal structure of aromatase has opened the door for investigating the dynamics by high resolution atomic/coarse-grained simulated models, such as molecular dynamics (MD) simulations and normal mode analysis (NMA). NMA proves to be a very powerful tool to gain insights into the protein dynamics at a reasonable resolution (heavy atoms or C**α**) at much less computational costs [4]. NMA in combination with elastic network (EN) model [5] has been developed for studying protein flexibility and dynamics [6], [7], [8], [9], [10], [11], [12], [13], [14], [15], [16], [17]. Due to the simple harmonic nature of the potential, the methodology is valid only in proximity to equilibrium and unable to model energy barriers and multiple energy minima. Nevertheless, it has been proven to yield the slow normal modes just as effectively as those from complicated forcefields with specific non-linear terms [12], [13]. The collective motions of a protein at the low-frequency spectrum are correctly correlated with the observed protein conformational changes upon ligand binding or protein-partner association [17].

In this paper, we present the results from EN-NMA on the membrane-free and membrane-integrated monomers and the crystallographic dimer and trimer of aromatase. We show that two major intermolecular modes of motion are responsible for alternations in the observed quaternary association of aromatase that could be utilized for its endoplasmic reticulum (ER) membrane integration. The two major intramolecular normal modes in the monomer are likely to be responsible for the active site access channel “breathing”. The root mean square fluctuation (RMSF) from EN-NMA provides a measure for the intrinsic molecular flexibility and the analysis elucidates the rigid core structure of aromatase, regardless of its self-association and membrane integration.

## Results

### EN-NMA of crystallographic aromatase oligomers

**Figure 1:**
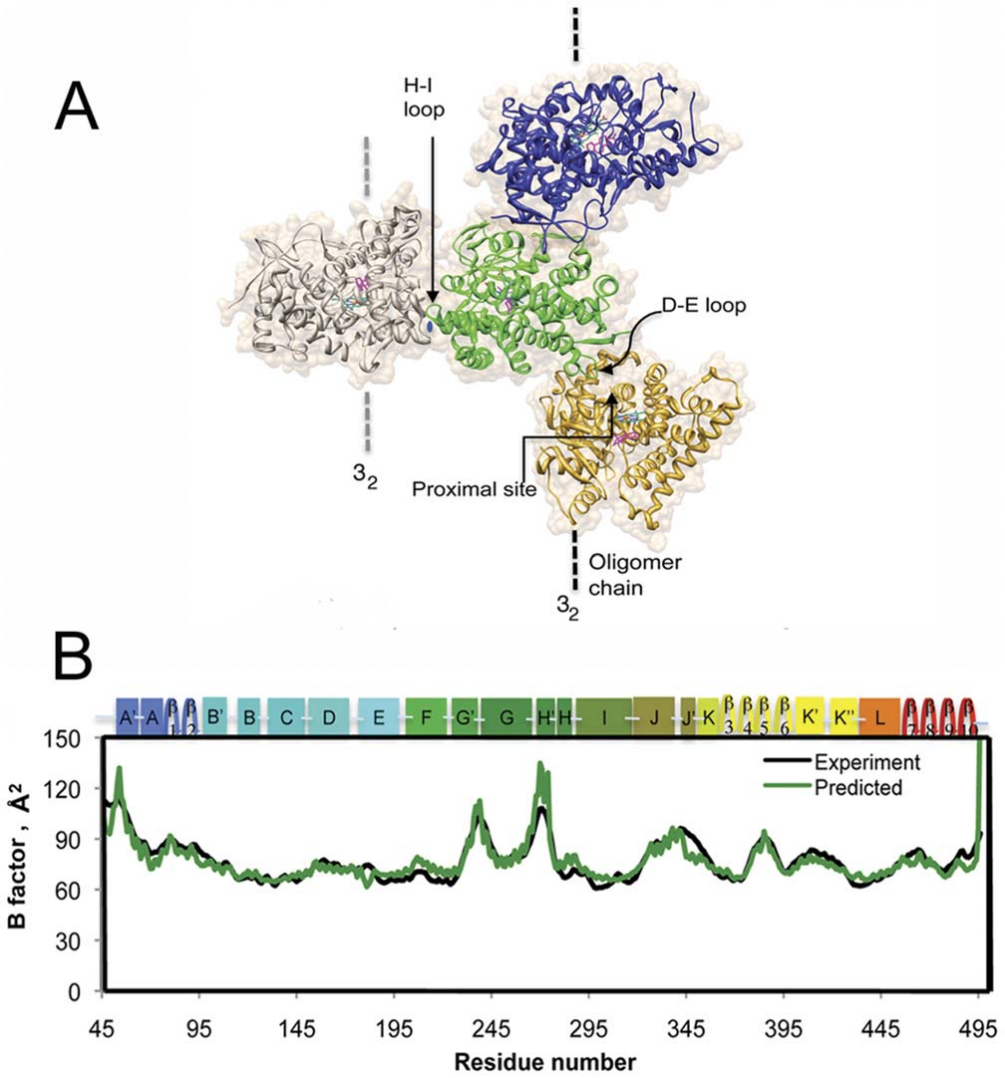
The crystallographic tetramer of aromatase and validation of the C_α_ normal mode analysis against crystallographic B factors. A, three aromatase monomers from one oligomer chain (ribbon diagram colored blue, green and orange) in contact through the H-I loops with another monomer (gray) from the neighboring chain. B, the computed B-factors of C_α_ of the central monomer (green line), simulating the closely packed aromatase in crystals, are compared well with those from X-ray data (black line). The substrate and heme group are represented by stick drawings. The former is colored in magenta while the latter is rendered in element colors: cyan, C; red, O; blue, N; brown, Fe. The coloring code and the atoms and bonds representations are the same in all figures unless otherwise noted.

A tetramer is built using the crystallographic symmetry (Fig. 1A) and then subjected to normal mode analysis. Within the tetramer, the central green monomer is found to have the smallest amplitude of displacement in the first two slowest modes, indicating that its global mobility is constrained by the head-to-tail association and crystal packing. Other three monomers display higher mobility because they are devoid of the crystallographic constraints, or the periodic boundary conditions. Taking mode 7 as an example, two regions with distinctly different mobility are clearly visible. The inner region of the central monomer and its vicinity are much less mobile than the outer region of the blue, gold and gray monomers (Fig. S1A, Supporting Information).

The B-factors of Cα (B-C_α_) are computed from the mean square fluctuation (MSF), for the green monomer and compared with the X-ray B-factors (Fig. 1B). The two agree with each other for a wide range of residues except for the termini. When compared with the X-ray B-factors, the B-C**_α_** factor profiles of the three outer monomers are significantly larger and exhibit substantial variations, unlike the inner green monomer (Fig. S1B, Supporting Information). The fluctuation patterns for the regions responsible for crystal packing and head-to-tail binding for these three monomers are dramatically different from the X-ray B-factors. The variations in global mobility and change in residue-fluctuation patterns are correlated well with the crystal contact interactions (see details in Supporting Information).

**Figure 2:**
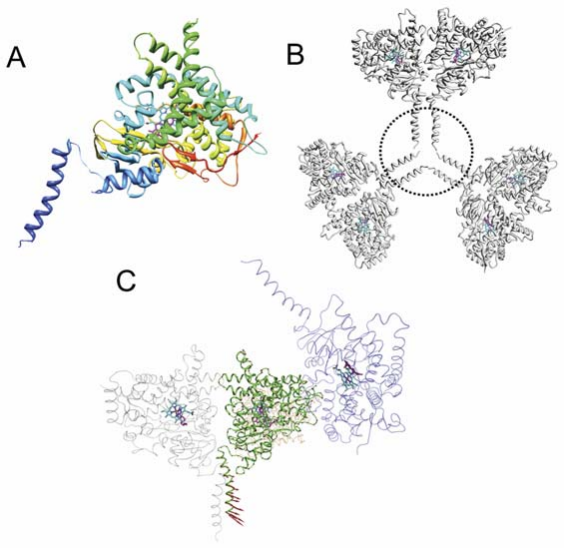
Ribbon diagrams showing the structure of (A) aromatase monomer with the N-terminal helix, (B) the observed void in the crystal and (C) the large collective motion of the N-terminal helix of the central monomer in the slowest normal mode. The dotted circle in (B) indicates the region of lowest electron density in the crystal, presumably a channel of solvent/detergent that also encompasses the dynamically disordered N-terminal helices. The eigenvectors were scaled by a factor of 200 for visualization purposes. The eigenvector arrows in (C) represent the relative amplitude and direction of the associated C_α_ atoms of the central monomer. The same eigenvector representation has been followed in Figs. 3 (A) to (D) and Fig. 4 (A).

A monomer with the N-terminal helix is shown in Fig. 2A and the formation of large voids, the regions of lowest electron density, is observed in the crystal where the N-terminal helices reside (Fig. 2B). The monomers of the crystallographic tetramer are packed in the same way as those in the absence of the N-terminal helices (Fig. S2, Supporting Information). Interestingly, the motion of the N-terminal helix is found to be consistent with the crystallographic symmetry of the molecules in the crystal (Movie S1, Supporting Information). The motion of the central monomer is highlighted in Fig. 2C, showing that its N-terminal helix has the largest eigenvectors among the entire monomer.

### Collective motion in membrane-free and membrane-integrated aromatase monomers

**Figure 3:**
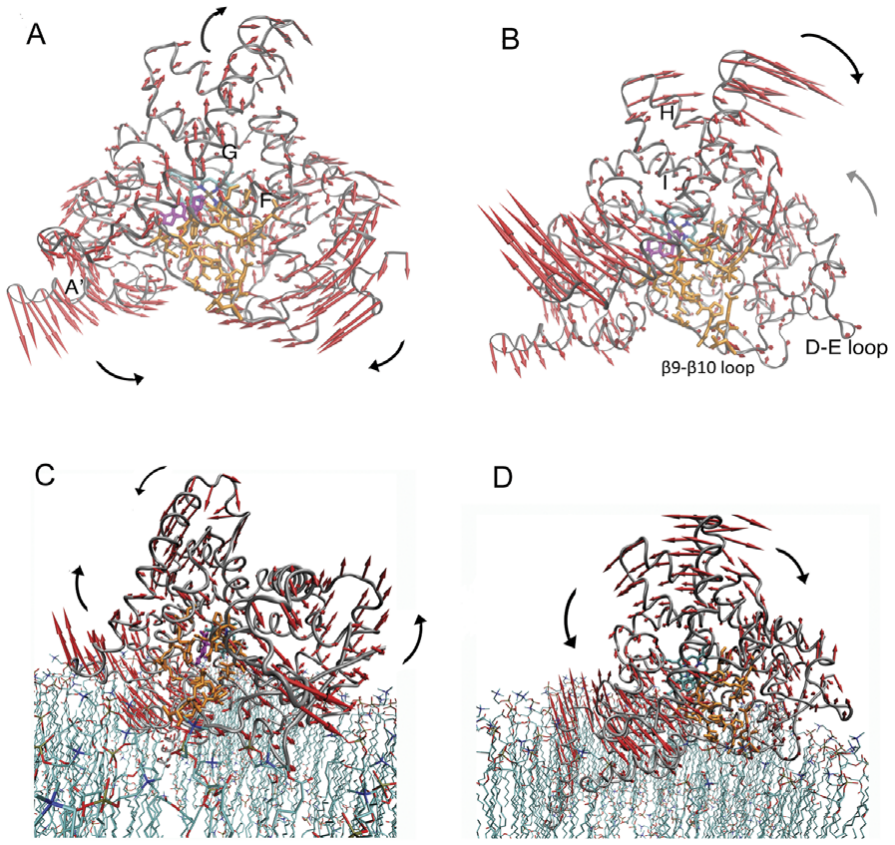
Intramolecular modes of motion of a membrane-free monomer and a membrane-integrated monomer by normal mode analysis. A, the three moving parts of a membrane-free monomer in the mode 7 producing the hinge-bending motions with the hinge at the active site access channel. B, two moving parts in mode 8 contributing to a twisting motion with the access channel at the interface. C and D, two internal normal modes (modes 19 and 17) show the intramolecular bending and twisting motions for a membrane-integrated monomer. The residues of the access channel are represented by sticks and rendered in orange color. The residues include Arg192, Ile217, Gln218, Phe221, Asp222, Ala225, Pro308, Asp309, Thr310, Ser312, Val313, Val369, Ile474, Ser478, Leu479, His480, Pro481, Asn482, Glu483 and Thr484. The eigenvector arrows are in the same indication as in Fig. 2 (C) while the large arrows depict the direction of collective motions.

The EN-NMA for a membrane-free monomer reveals interesting internal motions from the two slowest normal modes (Figs. 3A and B). In mode 7, three moving parts of the structure are identifiable: two in the lower half of the molecule librating in the opposite directions and the third in the upper half rotating against the lower half (Fig. 3A and Movie S2A, Supporting Information). The membrane-integrating N terminus and its vicinity form the first part, the C-terminal loop regions the second, and the segments above the active site access channel the third. The access channel residues are at the borders of these three moving parts. The movements of each pair produce the so-called “hinge-bending” motion [18], [19] with the common hinge being at the access channel. In mode 8, the front half of the molecule librates against the back, forming an intramolecular twisting motion with, again, the access channel at the interface (Fig. 3B and Movie S2B, Supporting Information).

When the monomer is embedded in the lipid bilayer, motions similar to those in the membrane-free monomer are observed within the monomer but at a higher frequency. Due to interactions at the membrane-protein interfaces, the hinge-bending motion (the mode 19) has reduced amplitudes for the membrane integrating regions (the helices A′ and A, and the C-terminal β7–β8 and β9–β10 loops) (Fig. 3C and Movie S3A, Supporting Information). Instead, the F-G loop and its vicinity have enhanced amplitudes and the C-terminal loop regions librate against the membrane, different from the movement in a membrane-free monomer. The twisting motion (the mode 17; Fig. 3D and Movie S3B, Supporting Information) exhibited is similar to that in the membrane-free molecule. The N-terminal helix is associated with the motions in the rear half of the molecule in Fig. 3D. It is noteworthy that the C-terminal loops are relatively stationary in both modes.

However, the three slowest modes, modes 7, 8 and 9, for the membrane-embedded monomer are unique and different from the above hinge bending and twisting motions. The former two are back-forth and left-right bending oscillations, respectively, and the latter is a twisting motion (Movie S4A, B, C, Supporting Information). The two bending modes result in rocking of the cytoplasmic domain of aromatase at the lipid interface in two directions. Twisting appears to be a counterclockwise, winding motion of the entire cytoplasmic domain about a vertical axis while keeping the N-terminal trans-membrane segments relatively stationary. As a result, the heme/active site region moves in and out of the lipid interior.

### Slow modes of crystallographic oligomers

The two slowest normal modes 7 and 8 are basically rigid body rotations against each other when only the green-gold dimmer is considered (Fig. S3, Movie S5A and B, Supporting Information). In the process, the region above the D-E loop including helix J, β7, β10, and the β7–β8 loop of the green monomer, and the heme-proximal cavity region constituted by helices B′, C, H, H′ and J-K loop of the gold monomer move back and forth to each other. These movements lead to the simultaneous opening/closure of two head-to-tail extended regions formed by the neighboring monomer pairs.

**Figure 4:**
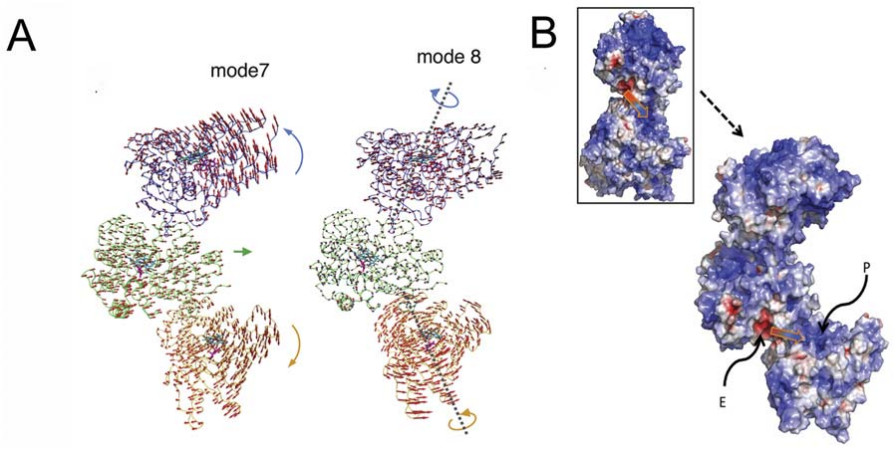
Intermolecular motions of the aromatase trimers from normal mode analysis and their complementarity with electrostatic interactions. A, two slowest normal modes in aromatase trimer. The dotted lines designate the rotational axes. B, electrostatic potentials mapped on the van der Waals surfaces of a trimer in a color scale red to blue representing a potential scale from −7*k*T/e to 7*k*T/e. The P and E sites, adjacent to the head-to-tail binding interfaces of the oligomers, correspond to the positively and negatively charged cavities, respectively. The arrow points roughly along the electrostatic potential gradient from negative to positive potentials. The orientation of oligomer is roughly the same in both panels. The inset shows the second dimer interface hidden from view.

These slowest normal modes are maintained within the crystallographic blue-green-gold trimer (Fig. 4A). In mode 7, the blue and gold monomers move away from/toward each other, while the green monomer undergoes a small back and forth translation (Movie S6A, Supporting Information). This movement consists of two asymmetrical hinge-bending motions, one between the blue and the green monomers and the other between the gold and the green. In mode 8, two twisting motions are formed through the rotation of either blue or gold monomer against the nearly stationary green monomer. The rotation axes are roughly the lines linking the centers of the mass with their respective head-to-tail binding sites (a cross-section view in Movie S6BI and a plan view in Movie S6BII, Supporting Information). Interestingly, these two intermolecular motions are preserved in a trimeric aromatase even in the presence of the fourth gray monomer, simulating the crystal-packing environment (Movie S7A, BI and BII, Supporting Information). Nevertheless, examination of the higher frequency modes confirms the presence of bending and twisting modes similar to the ones in a membrane-free monomer, only muffled due to intermolecular association.

The electrostatic potentials of a dimer and a trimer are calculated and mapped on their van der Waals surfaces (Fig. 4B). In a dimer, two major groove sites form an electrostatic potential gradient near the head-to-tail binding site, site “*E*” with negative electrostatic potentials on the upper monomer and site “*P*” with positive electrostatic potentials on the lower. Nine negatively charged side chain residues, Asp 186, Asp197, Asp209, Asp222, Asp482, Glu177, Glu210, Glu483 and Glu489, contribute to the negative electrostatic potentials at the *E* site, and about six positive charges from Lys 142, Lys352, Lys440, Lys448, Arg 145, Arg375 and the heme group form the positive electrostatic potentials at the *P* site. In a trimer, a pair of such *E* and *P* sites is present at each head-to-tail binding site (Fig. 4B). The electrostatic potential gradient could be of interest here and could influence the intermolecular motions. The direction of intermolecular motions would be favorable along the gradient, but unfavorable against it.

We also probed by computational approaches other possible oligomeric interfaces that aromatase monomers may utilize in solution. An overwhelming majority of the models thus obtained showed the crystallographically observed interface as the inter-monomer interaction surface. Furthermore, the results also suggested considerable flexibility in the D-E loop-to-heme proximal cavity association within the interface (Text S1 and Fig. S4, Supporting Information).

### Fluctuation in aromatase

**Figure 5:**
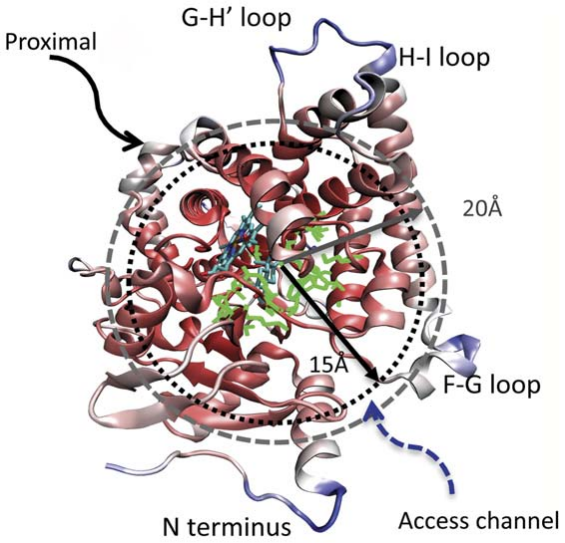
The flexibility of a membrane-free aromatase monomer. A ribbon diagram rendered in the color scale red to dark blue representing 0.4 to 2.7 in the relative root mean square fluctuation (RMSF); the atoms and bonds of the catalytic cleft are rendered in green; the solid arrow points to the heme-proximal cavity and the dashed arrow roughly the location of the access channel. Three layers are marked with dashed lines in a radius of *r*<15 Å, 15 Å≤*r*<20 Å and *r*≥20 Å, starting from the center of the substrate.

The Cα-RMSF of an aromatase monomer (Cα-RMSF) is calculated and visualized in a rendered ribbon diagram where red represents the lowest RMSF (at the heme group), and blue the highest RMSF (at the H-I loop) (Fig. 5). The H-I, D-E (not shown), G-H′ and F-G loops (including the short helix G′ and its connecting loops to helixes G and F) are quite flexible, but the inner core, defined as a spherical region within a radius of 15 Å from the center of substrate, is very rigid. The catalytic cleft is at the center of the core. The average RMSFs, either in the absence or the presence of the substrate, are calculated over four distinct regions: heme, the catalytic cleft, the heme-proximal cavity and the active site access channel, and also over three layers of interest within the aromatase molecule: the inner core (radius≤15 Å), middle-layer (15 Å<radius≤20 Å) and outer layer (radius>20 Å) (Fig. S5A, Supporting Information). Heme has the lowest RMSF, 0.47, followed by the catalytic site 0.52 and the inner core 0.65 in the presence of the substrate. They are all well below the average fluctuation (RMSF = 1) of the molecule. The putative access channel has a higher RMSF of 0.74 when compared with heme, the catalytic cleft and the inner core, probably due to some of its constitutive residues, such as Pro481, Asp482, Glu483 and Thr484 from the β9–β10 loop, are either lining the channel or bordering the lipid interface. A modest fluctuation, an RMSF of 0.92, has been found in the proximal cavity, most of whose constitutive residues are from the 21-residue long K″-L loop but stabilized by the heme group through coordination with Arg435 and the Cys437 ligation. The fluctuation of the middle layer is about 10% below the average fluctuation of the molecule and that of the outer layer is the largest (∼35% above the average fluctuation).

Although the substrate is in direct contact with the catalytic cleft residues [1], only a marginal increase of 0.04 in the RMSF is found in the absence of substrate (Fig. S5A, B, Supporting Information). It appears that removal of the substrate does not significantly affect the rigidity of the catalytic cleft. Interestingly, however, similar small but consistent increases in RMSF in the heme-proximal cavity, the access channel and the inner core are observed on substrate removal, but not in the middle or outer layers. Therefore, the stabilizing effect of substrate binding on the protein rigidity is rather small due to the compact nature of the aromatase molecule, and is limited to the inner core, not exceeding a 15 Å radius. Furthermore, the heme moiety could primarily be responsible for the overall rigidity of the catalytic cleft resulting from stabilization of the side chains, such that the integrity of the functionally active enzyme is maintained even in the absence of the substrate.

The Cα-RMSFs calculated for a membrane-integrated aromatase monomer (Fig. S6A, Supporting Information) show that the catalytic cleft has similar low fluctuations as the heme, followed by the access channel and the proximal site. Notably, the fluctuations of these four segments have an order similar to those of a membrane-free monomer. The N-terminal helix has relatively higher fluctuations due to its location away from the body of the molecule. Thus, the rigid core structure of aromatase is intrinsic and independent of its membrane integration.

To evaluate possible impact of the side chains on these results, we compare the RMSFs of the catalytic cleft, the inner core, the middle layer and the outer layer with those obtained from all-heavy atom NMA (Fig. S6B, Supporting Information). The results agree with each other within 0.09 RMSF, implicating that the side chain mobility is correlated with the main chain flexibility in the monomeric aromatase assuming that it does not undergo any large structural transition.

In addition, calculations of the fluctuations of aromatase oligomers show that the oligomerization does not deteriorate the rigidity of the active site (Fig. S7 and Text S2, Supporting Information).

## Discussion

### Model validation in crystal environment

The EN-NMA is attractive because it has the capability to identify the slowest internal modes of protein that are important for biological functions [12], [17]. Our calculations on a tetramer validate the applicability of NMA. The computed B factors of the crystallographic central monomer agree well with the experimental X-ray B factor data. Moreover, the results show that the method is sensitive to inter-monomer association and crystal packing interactions. The RMSF peaks disappear at the tail (the D-E loop and vicinity) and the head (the K helix, J′-K and K″-L loops) regions upon head-to-tail association in which the helix, loops and strands embed into the protein interior (Fig. S1, Supporting Information). The D-E loop vicinity includes the β8 strand from sheet 3, and β7 and β10 from sheet 4. These results also confirm that the shape of the tail of one monomer compliments the proximal cavity of the next in an aromatase oligomer and the self-association is stabilized by intermolecular interactions. It is conceivable that the heme moiety plays a major role in the stability of the proximal cavity and hence influences the oligomerization. The peaks at the H-I loop interface disappear due to the crystal packing constraints.

Moreover, the NMA of crystallographic tetramer shows that the N-terminal helices have large mobilities. These motions, however, appear not to break the crystallographic symmetry or interfere with intermolecular packing. This could explain why the N-terminal region of the molecule appears dynamically disordered in electron density maps.

### Complexation-induced rigidity

Self-association decreases the flexibility of a monomer in the oligomeric aromatase at the head-to-tail binding site and its vicinity. The result is similar to the phenomenon reported in the analysis of Ras-Raf using a molecular framework approach and MD simulation [20]. As we have also observed in the aromatase trimer, the regions distant from the binding sites become more flexible upon aromatase self association, the perturbation generated from aromatase self association can propagate from a binding site to remote regions by alternating the dynamic network of interactions in proteins. The translational and rotational degrees of freedom of the monomers in an aromatase oligomer are reduced due to monomer-monomer binding. The “freezing-out” of possible multiple structures of an oligomer upon binding results in loss of configurational entropy, but it could be compensated by the entropy gain from the increase in flexibility of the distant regions away from the binding site (Fig. S6A, Supporting Information) as proposed by Steinberg et al. [21]. Entropic contribution from the increased flexibility is believed to be a dominant factor in the free energy of protein-protein association [22].

The fluctuations of both the access channel cavity and proximal site relative to heme reduce on integration into the membrane. The RMSF ratio decreases from 1.77 to 1.33 for the proximal site and from 1.45 to 1.14 for the channel cavity, while the active cleft remains roughly the same. This could be due to the fact that the active site residues are located away from the membrane surface, whereas some of the access channel resides and the loop residues of the proximal site interact with the lipid bilayer. However, these predictions need further validation by site-directed mutagenesis in reconstituted membrane and/or cell-based activity assay on the mutant enzymes.

Furthermore, the observed reduction in the mobility of the membrane-associating C-terminal loops could result in enhanced stability and optimal alignment of the active site access channel for steroidal passage through the lipid bilayer.

### Possible pathway for a crystallographic oligomer to integrate into membrane

**Figure 6:**
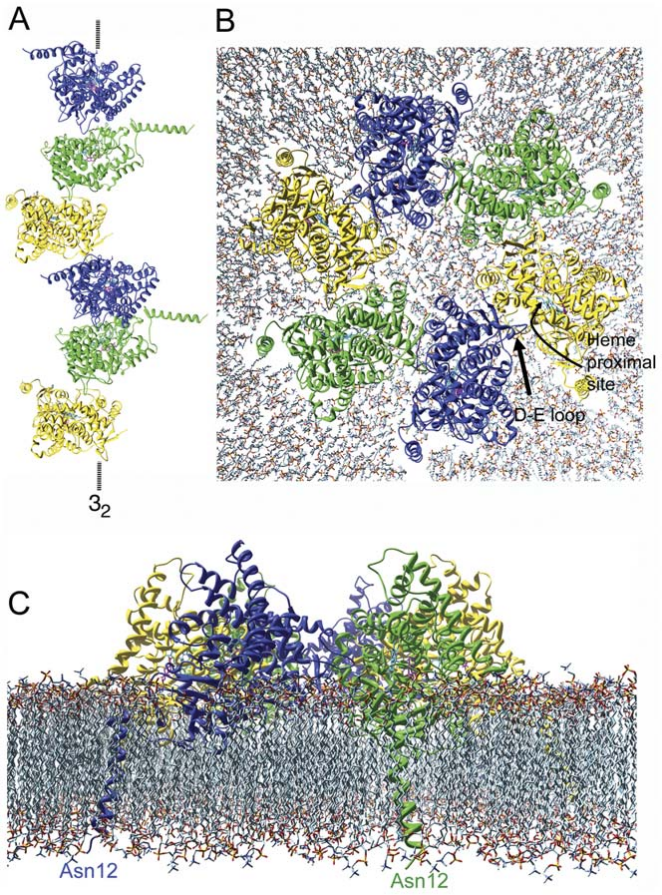
Transformation of a linear to a circular hexamer that has the correct membrane insertion topology. A, a linear hexamer (a chain of two units of trimers) related by the crystallographic 3_2_ screw symmetry. B and C, the plan and cross-section of a circular hexamer inserted within a lipid bilayer.

A valid aromatase oligomer topology should be amenable to integration into the ER membrane. We have used two linear trimer units (Fig. 6A) to model a membrane-integrated circular hexamer (Figs. 6B and C) by a process described in the Materials and Methods section (see below). A combination of the twisting and hinge-bending motions shown in Fig. 4A could adjust the quaternary association along the lowest energy landscape [18]. Electrostatic interaction between the “*E*” site of the “tail” monomer and the “*P*” site of the “head” is presumed to play a role in driving the movements for the quaternary structural changes. The modeling suggests that a circular oligomer thus formed would use a similar loop-to-proximal cavity link as that used by the polymeric chain in the crystal. The N-terminal helix of each monomer penetrates into and across the lipid bilayer with its end in the lumen side.

The size of a circular oligomer may vary depending on aromatase concentration. An open passage in the membrane is just created for each monomer after membrane insertion and each passage is connected to the access channel of each molecule (same as for a monomer in Fig. S6A, Supporting Information). Organizations such as cyclic hexamers (size ∼14 nm), octamers (∼18 nm) and even higher orders could be modeled in this way. In the resting state, oligomerization could be a means of protection of integrity of the proximal site and/or from undesirable effects at the site, such as non-specific actions of redox agents, and phophorylation of Tyr 361 [23]. A likely scenario is that the monomers are replaced by the CPR molecules for the electron transfer reaction and aromatization to proceed.

### Flexibility and dynamical motion: relevance to biological function

The N-terminal helix, novel to the P450 structures elucidated thus far, is the most mobile and flexible structural element identified. The F-G loop is the next most flexible region in the aromatase structure (Fig. 5) that is not significantly influenced by self-association and membrane integration. The F-G loop flexibility was previously reported to be one of the common features of cytochrome P450s 2B4 and BM-3 with functional relevance to enzymatic reactions [24]. The flexible loop undergoes an open/close motion that allows the steroids to enter into or leave from the active site through the access channel [1], [25]. Our results provide new support to this notion. Furthermore, the NMA of a monomer reveals that the access channel could serve as a hinge for intramolecular bending and an interface for twisting motions. These motions, together with the intrinsic flexibility of the access channel, are likely to contribute to channel “breathing”, opening and closing of the channel mouth and the cavity, perceived necessary for entry and exit of steroids to and from the active site.

The hinge bending and twisting motions at the access channel hinge/interface are also present in the lipid-embedded aromatase, but at a higher frequency. The membrane penetrating areas, such as helices A′ and A, have reduced amplitudes, owing perhaps to dampening of the oscillation by surrounding lipid molecules. However, the twisting motion is similar to the membrane free molecule, which suggests that twisting could be more closely related to a functional aromatase in vivo. Interestingly, the N-terminal helix motion does not coordinate with either of these two movements; instead, it is associated with the rear half of the molecule, suggesting that membrane integration of the N-terminal helix may have roles different from “breathing” or steroid passage, perhaps in intramembrane stabilization or CPR coupling. One of the slowest modes of the membrane-embedded aromatase suggests a periodic movement of the active site region deeper toward the lipid interior. Such a motion could be associated with the enzyme's substrate sequestration and/or product release phases of the catalytic cycle.

Two slowest modes at the interface of the head-to-tail association are intermolecular rigid-body hinge bending and twisting motions. They provide the flexibility for the aromatase molecules to reorganize themselves retaining the interface in order to form an oligomeric structure. Our data suggests that such reorganization and reorientation are necessary to position the trans-membrane helices and regions on the same side of each monomer for the oligomer as a whole to penetrate the lipid bilayer. The driving force for this interfacial motion could be drawn from the electrostatic potential gradient between the electronegative “*E*” site of the D-E loop region of one monomer and the electropositive *“P”* site of the heme-proximal region of the other. The heme-proximal electropositive *“P”* site of aromatase has been proposed to be critical for electron transfer by the FMN moiety from CPR [26]. The observed flexibility of the intermolecular interaction from this work suggests that the FMN moiety of CPR could bind at the interface, either by flexing the head-to-tail organization for a three-way binding or by competitively replacing an aromatase monomer.

One of the most important biological implications of our computational results is the corroboration that intermolecular contacts and flexibility observed in the crystal structure could be utilized into a higher order organization of aromatase that has the correct topology for membrane integration. Aromatase molecules function in the ER membrane and recent results suggest that the enzyme is multimeric when embedded in the lipid bilayer [3]. Our data derived from the crystal structure and flexibility calculations show a mechanism by which this could be done, maintaining the heme-proximal site orientation accessible for CPR coupling. Furthermore, the computational result on free monomer to monomer docking suggest that head-to-tail organization observed in the crystalline aromatase is the most favored interface albeit with a good deal of flexibility. Taken together, these results provide new atomic level insights into the form, function and flexibility of an oligomeric aromatase previously envisioned in the literature.

Lastly, the present aromatase atomic model for the first time shows the N-terminal trans-membrane helix, based partly on weak experimental electron density map not previously modeled, and partly on the crystal packing constraints. Although other microsomal P450's are known to have similar trans-membrane segments, aromatase in particular has longer and more pronounced membrane-integrating regions, as the crystal structure and sequence comparison suggest [1], [26]. Modeling of this helix, and its juxtaposition in relation to other membrane integrating A′ and A helices, as well the C-terminal membrane associating areas all reaffirm the previously proposed notion that the opening to active site access channel rests just inside the lipid bilayer enabling easy passage of the highly hydrophobic steroidal substrate and product. The N-terminal helix appears to project out into the lipids via an extended peptide segment with residues Tyr41 to Gly49 away from the main enzyme structure. This is suggestive that the trans-membrane segment probably plays roles not directly associated with the enzyme catalysis, but in the aromatase-CPR interaction through the CPR's transmembrane segments. Indirectly, however, such “tethered” membrane anchoring may be crucial for the added flexibility of the business end of the molecule, as the slowest modes suggest. The observed dynamical disorder of the N-terminus in the X-ray data is simply a reflection of the fact that once separated from the membrane it dangles harmlessly away from the main structure and without any interference with the stability of the functional enzyme. The current modeling of the N-terminal helix and its vibrational modes within the apparent “void” of the aromatase crystal (see “Calculations under crystal packing conditions” in MATERIALS AND METHODS) is a confirmation that its enhanced mobility persists and accommodated in the crystalline state.

We show that the major normal modes in aromatase oligomers are inter-monomeric rigid body motions with the D-E loop to proximal site association as the interface and that this interface is directly linked to catalytic function of the enzyme. It is, therefore, likely that suitable small molecules binding at the interface would interfere with the CPR coupling, oligomer formation and/or its membrane integration. Such non-active site directed compounds could constitute a new class of aromatase inhibitors.

## Materials and Methods

### EN-NMA

The detailed description and recent reviews of the C**α**-NMA method can be found in literature [7], [12], [13], [15]. In this work, a single parameter potential was used as proposed by Tirion [5]. The building block approximation so called rotation-translation-block (RTB) [8] method is employed to speed up our calculations and reduce the computational limitation of a large system. As mentioned by Bahar [10], [12] and Tama [8], this approximation has very little influence on slow modes, particularly for a large protein complex where the functional domains are expected to be large.

The oligomer coordinates were generated by the crystallographic symmetry operations using Coot [27] and the crystal structure of the human placental aromatase monomer [1], PDB code: 3EQM. NMA was implemented using the elNémo web-server [28]. The smallest system of study consisted of one monomer (452 residues) and there were 1940 residues (including the N-terminal helices) in the largest system consisting of a tetramer. The connectivity cutoff distance, used in pairwise Hookean potential between nodes, was tested in a range of 8∼16 Å. A cutoff of 10 Å was selected for monomer and the default of 8 Å for oligomers after scaling with the experimental B factors. The block size was chosen by default, but could be varied with the system size. For convenience of visualization, the eigenvectors were scaled by a factor of 200. The eigenvector arrows in Fig. 2(C) represent the relative amplitude and direction of the associated C_α_ atoms of the central monomer. The same eigenvector representation has been followed in Figs. 3 (A) to (D), Fig. 4 (A), and in Supporting Information (Fig. S1 and Fig. S3). The normal mode models were computed with a given perturbation amplitude in the direction of a single normal mode. Here the perturbation range was from −100 to 100 with a step size of 20 [28]. The motion in supporting movies (Movies S1, S2, S3, S4, S5, S6, S7) was generated under perturbation; it could, therefore, be exaggerated when compared with equilibrium fluctuation.

The N-terminal helix was not included in the calculations except for the protein in complex with the membrane or wherever noted. It is seen that the movement of the N-terminal helix are predominant in the absence of membrane among the collective motion modes that have a low frequency and large amplitude. The usage of a truncated aromatase model is found more efficient than that with the N-terminal helix in the dynamics study of oligomers.

The frequency was normalized relative to the lowest mode frequency in all our calculations. The frequencies of modes 7 and 8 of a dimer were 1.00 and 1.09, and those of a free monomer were 1.00 and 1.04. Taking the slowest frequency to be 2.5 cm^−1^, the frequencies of the first 20 slowest modes in the system of this study are in the range 2.5–15 cm^−1^. Therefore, the time scale for the slowest modes range from a few picosecond to the order of 10 picosecond, in agreement with reported collective motion in proteins [18].

### Calculations under crystal packing conditions

In the space group P3_2_21, the head-to-tail oligomers are formed about the crystallographic three-fold screw axis and packed in the crystal about a crystallographic 2-fold rotation axis perpendicular to the 3_2_ screw axis (Fig. 1A). A head-to-tail intermolecular interaction among aromatase molecules is mediated via a surface loop between helix D and helix E of one aromatase molecule penetrating into the heme-proximal cavity of the next, thus forming a polymeric aromatase chain (Fig. 1A). Two oligomer chains form crystal contact through hydrogen bonding and salt bridge interactions via the H-I loops. For the system of four interconnected crystallographic monomers in Fig. 1A employed in the calculation, the crystal-packing environment was preserved for the central green monomer. However, the adjacent blue and gold monomers from the same polymer chain and the gray from the neighboring chain each had only one association, unlike the crystal environment.

The calculations were repeated for the crystallographic tetramer with the N-terminal helices (Fig. S2, Supporting Information). A putative atomic model, consisting primarily of an α-helix, for the N-terminal missing residues Asn12 to Thr44 is built using the partially visible weak experimental electron density [1] (Fig. S8, Supporting Information), and restrictions of the crystallographic 2-fold rotation axis, which the two symmetry-related helices approach. The modeling was also guided by the fact that a helix between Ile13 and Tyr40 would traverse the lipid bilayer, positioning Asn12, a potential glycosylation site, in the ER lumen. Interestingly, N-terminal helices line up about the 3_2_ symmetry axis within the crystal in the space that constitutes the largest void (a region of lowest electron density in the crystal), a channel of dynamically disordered solvent and detergent, thereby providing some rationale as to why the N terminus is disordered.

### Modeling of aromatase monomer in lipid bilayer

It is known that phosphatidylcholine is the major lipid composition in ER membrane [29], [30], [31]. For simplicity, a bilayer model of 1-palmitoyl-2-oleoyl-phosphatidylcholine (POPC) is employed to represent the ER membrane. The coordinates of the phospholipids were generated with the builder module in the VMD package [32] and the membrane has a size of 80 Å
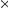
80 Å. The aromatase molecule was then aligned against the membrane according to the hydrophobic property of the protein and their topology, as described earlier [1]. The N-terminal helices, up to helix A, traverse into the bilayer with the Asn12 in the lumen side. The C-terminal loops, such as β7–β8 and β9–β10 loops are embedded into the lipids. The structures of protein and membrane were then merged with the VMD package by eliminating the lipid molecules that overlap with the protein. The complex were finally subjected to energy minimization at the protein-membrane interface with fixed backbone of the protein and lipid molecules in Molecular Operating Environment (MOE, 2009.10), Chemical Computing Group, Montreal Canada [33]. There are 160 lipid molecules and one aromatase molecule in a total of about 12,000 heavy atoms in the system.

### Characterization method of aromatase flexibility

Root mean square fluctuation (RMSF) was used to characterize protein flexibility. The mean square fluctuation (MSF) of the *i*th node, 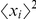, could be determined from the normal modes [13], [16]

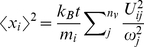
(1)where *k*_B_ is the Boltzmann constant, *T* the system temperature, *m*_i_ the mass of the *i*th node, *U*_ij_ the eigenvector of the *i*th node with the frequency *ω*_j_, and *n_v_* is the number of modes considered. An accurate evaluation of MSF was achieved from the average of the 100 slowest normal modes. The B factor of each node was calculated using the relationship of
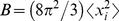
(2)and further rescaled by an origin shift and a scale factor multiplier. The former is necessary to account for the contribution from rigid body motion implicit in the X-ray B-factors [14] and the latter is used to match the X-ray data. NMA was carried out with variables such as temperature, atomic mass and potential energy, in reduced units, so that the unit of MSF was also reduced. Here, the flexibility of a node was characterized by the relative RMSF of the node to the mean value of the system, i.e. the computed RMSF was in reference to system of study. For simplification, RMSF used in the paper refers to the relative RMSF unless otherwise noted. The residue RMSF was given as the average over the backbone atoms in all heavy-atom NMA, and as the value for the C**_α_** atoms in *C* **_α_**-NMA. Flexibility of a region of interest was depicted by the average of residue-RMSFs over this region. Because MSF is in reduced unit, the calculated B factors (from Eq. 2) were scaled to the experimental B factor data. Prior to scaling, the calculated MSF of each node was reasonably up shifted away from the origin to account for the translational and rotational rigid body motions in the lattice cell.

### Calculation of the electrostatic potentials of aromatase oligomer

The software Adaptive Poisson-Boltzmann Solver (APBS) [34] as a plug-in to Pymol [35], was used to calculate the electrostatic potentials of the aromatase dimer and trimer. The coordinate files of these crystallographic oligomers were prepared in the same way as those in NMA. The electrostatic interactions between solutes in solvent media were evaluated by solving the Poisson-Boltzmann equation (PBE) [36], a popular continuum model. The parameters, such as grid dimension, length and spacing, etc. were setup in default values as suggested by the program. The calculated electrostatics were visualized with Pymol by mapping them on the van der Waals surface of protein molecules rendered in a color spectrum from red to blue representing the scale from −7*k*T/e to 7*k*T/e.

### Modeling a hexamer on membrane

**Figure 7:**
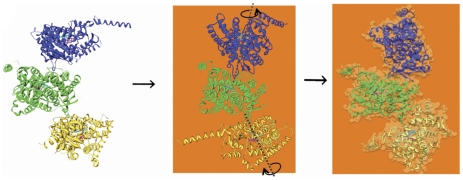
A proposed mode of utilization of intermolecular motions for membrane integration by a unit of crystallographically related trimer. The indicated rotational motions about the dotted axes align the N-terminal helices. The orientation of the oligomer is roughly the same as in all panels of Fig. 4.

The atomic model of aromatase with the N-terminal helix was used to build by collapsing a linear chain of two units of crystallographically related trimers (Fig. 6A) into a circle in which all N-terminal helices have similar orientations (Figs. 6B and C). This was achieved primarily by rotating the each molecule pair roughly ±120° with D-E loop-in-proximal-cavity as the fulcrum as indicated in Fig. 7, such that the N-terminal helices all align on the same side of the hexamer. Some hinge bending and translational adjustments were made as well in order to form a symmetrical hexagon to avoid any steric violation. At the end, however, similar D-E-loop to proximal site contacts as observed in the crystal structure was maintained in all six monomer-to-monomer interfaces. Finally, the hexamer was built on a membrane (20nm
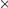
20nm) by the process described above (modeling of aromatase monomer in lipid bilayer).

## Supporting Information

Figure S1 The slowest normal mode of crystallographic tetramer of aromatase and the residue fluctuations against crystal contact interactions.
(TIF)

Figure S2 Ribbon diagrams showing the structure of a crystallographic tetramer.
(TIF)

Figure S3 Intermolecular motions of aromatase dimers from normal mode analysis.
(TIF)

Figure S4 Monomer-to-monomer docking computation.
(TIF)

Figure S5 The flexibility of a membrane-free aromatase monomer.
(TIF)

Figure S6 Membrane integration of aromatase and flexibility.
(TIF)

Figure S7 The flexibility of aromatase upon oligomerization.
(TIF)

Figure S8 Model for the N-terminal helix.
(TIF)

Text S1 Probing of other possible oligomeric structures by protein-protein docking computation.
(DOCX)

Text S2 Fluctuations in an oligomeric aromatase.
(DOCX)

Movie S1 Mode 12 of a crystallographic tetramer shows the motions of the N-terminal helices.
(MOV)

Movie S2 Two modes of intramolecular motion of a membrane-free aromatase monomer deduced from normal mode analysis: (A) hinge-bending mode and (B) twisting mode.
(MOV)

Movie S3 Two modes of intramolecular motion of a membrane-embedded aromatase monomer deduced from normal mode analysis: (A) hinge-bending mode and (B) twisting mode.
(MOV)

Movie S4 Three slowest modes of a membrane-embedded aromatase monomer: (A) and (B) bending and (C) twisting modes.
(MOV)

Movie S5 Two modes of intermolecular motion of the green-gold dimer deduced from normal mode analysis: (A) hinge-bending mode and (B) twisting mode.
(MOV)

Movie S6 Two modes of intermolecular motion of the blue-green-gold trimer deduced from normal mode analysis: (A) hinge-bending mode and (B) twisting mode.
(MOV)

Movie S7 Two modes of intermolecular motion deduced from normal mode analysis of the blue-green-gold trimer in the presence of the fourth gray monomer, simulating crystal packing conditions: (A) hinge-bending mode and (B) twisting mode.
(MOV)

## References

[1] Ghosh D, Griswold J, Erman M, Pangborn W (2009). Structural basis for androgen specificity and oestrogen synthesis in human aromatase.. Nature.

[2] Ghosh D, Jiang W, Lo J, Egbuta C (2011). Higher order organization of human placental aromatase.. Steroids.

[3] Praporski S, Ng SM, Nguyen AD, Corbin CJ, Mechler A, Zheng J, Conley AJ, Martin LL (2009). Organization of cytochrome P450 enzymes involved in sex steroid synthesis: protein-protein interactions in lipid membranes.. J Bio Chem.

[4] Phan G, Benabdelhak H, Lascombe MB, Benas P, Rety S (2010). Structural and dynamical insights into the opening mechanism of P. aeruginosa OprM channel.. Structure.

[5] Tirion M (1996). Large amplitude elastic motions in proteins from a single-parameter, atomic analysis.. Phys Rev Lett.

[6] Go N, Noguti T, Nishikawa T (1983). Dynamics of a small globular protein in terms of low-frequency vibrational modes.. Proc Natl Acad Sci USA.

[7] Brooks BR, Bruccoleri RE, Olafson BD, States DJ, Swaminathan S, Karplus M (1983). CHARMM: a program for macromolecular energy, minimization, and dynamics calculations.. J Comput Chem.

[8] Tama F, Gadea FX, Marques O, Sanejouand YH (2000). Building-block approach for determining low-frequency normal modes of macromolecules.. Proteins.

[9] Taly A, Delarue M, Grutter T, Nilges M, Le Novere N (2005). Normal mode analysis suggests a quaternary twist model for the nicotinic receptor gating mechanism.. Biophys J.

[10] Bahar I, Atilgan AR, Erman B (1997). Direct evaluation of thermal fluctuations in proteins using a single-parameter harmonic potential.. Fold Des.

[11] Kovacs JA, Chacon P, Abagyan R (2004). Predictions of protein flexibility: first-order measures.. Proteins.

[12] Bahar I, Lezon TR, Bakan A, Shrivastava IH (2010). Normal mode analysis of biomolecular structures: functional mechanisms of membrane proteins.. Chem Rev.

[13] Dobbins SE, Lesk VI, Sternberg MJ (2008). Insights into protein flexibility: The relationship between normal modes and conformational change upon protein-protein docking.. Proc Natl Acad Sci U S A.

[14] Soheilifard R, Makarov DE, Rodin GJ (2008). Critical evaluation of simple network models of protein dynamics and their comparison with crystallographic B-factors.. Phys Biol.

[15] Ma J (2004). New advances in normal mode analysis of supermolecular complexes and applications to structural refinement.. Curr Protein Pept Sci.

[16] Van Wynsberghe A, Li G, Cui Q (2004). Normal-mode analysis suggests protein flexibility modulation throughout RNA polymerase's functional cycle.. Biochemistry.

[17] Tama F, Sanejouand YH (2001). Conformational change of proteins arising from normal mode calculations.. Protein Eng.

[18] Brooks B, Karplus M (1985). Normal modes for specific motions of macromolecules: application to the hinge-bending mode of lysozyme.. Proc Natl Acad Sci U S A.

[19] Ma J, Karplus M (1998). The allosteric mechanism of the chaperonin GroEL: a dynamic analysis.. Proc Natl Acad Sci U S A.

[20] Gohlke H, Kuhn LA, Case DA (2004). Change in protein flexibility upon complex formation: analysis of Ras-Raf using molecular dynamics and a molecular framework approach.. Proteins.

[21] Steinberg IZ, Scheraga HA (1963). Entropy changes accompanying association reactions of proteins.. J Biol Chem.

[22] Forman-Kay JD (1999). The ‘dynamics’ in the thermodynamics of binding.. Nat Struct Biol.

[23] Catalano S, Barone I, Giordano C, Rizza P, Qi H (2009). Rapid estradiol/ERalpha signaling enhances aromatase enzymatic activity in breast cancer cells.. Mol Endocrinol.

[24] Poulos TL (2003). Cytochrome P450 flexibility.. Proc Natl Acad Sci U S A.

[25] Scott EE, He YA, Wester MR, White MA, Chin CC (2003). An open conformation of mammalian cytochrome P450 2B4 at 1.6-A resolution.. Proc Natl Acad Sci U S A.

[26] Hong Y, Li H, Yuan YC, Chen S (2010). Sequence-function correlation of aromatase and its interaction with reductase.. The Journal of steroid biochemistry and molecular biology.

[27] Emsley P, Cowtan K (2004). Coot: model-building tools for molecular graphics.. Acta Crystallogr D Biol Crystallogr.

[28] Suhre K, Sanejouand YH (2004). ElNemo: a normal mode web server for protein movement analysis and the generation of templates for molecular replacement.. Nucleic Acids Res.

[29] Zinser E, Sperka-Gottlieb CD, Fasch EV, Kohlwein SD, Paltauf F (1991). Phospholipid synthesis and lipid composition of subcellular membranes in the unicellular eukaryote Saccharomyces cerevisiae.. J Bacteriol.

[30] Brown DJ, Dupont FM (1989). Lipid Composition of Plasma Membranes and Endomembranes Prepared from Roots of Barley (Hordeum vulgare L.) : Effects of Salt.. Plant Physiol.

[31] Davison SC, Wills ED (1974). Studies on the lipid composition of the rat liver endoplasmic reticulum after induction with phenobarbitone and 20-methylcholanthrene.. Biochem J.

[32] Humphrey W, Dalke A, Schulten K (1996). VMD: visual molecular dynamics.. J Mol Graph.

[33] Chemical Computing Group (2009). Molecular Operating Environment. 2009.10 ed.

[34] Baker NA, Sept D, Joseph S, Holst MJ, McCammon JA (2001). Electrostatics of nanosystems: application to microtubules and the ribosome.. Proc Natl Acad Sci U S A.

[35] DeLano WL (2002). The PyMOL molecular graphics system.

[36] Fogolari F, Brigo A, Molinari H (2002). The Poisson-Boltzmann equation for biomolecular electrostatics: a tool for structural biology.. J Mol Recognit.

