# Supplementary figures and images for: Motion and Flexibility in Human Cytochrome P450 Aromatase
Source: PLoS One. 2012 Feb 27;7(2):e32565. doi: 10.1371/journal.pone.0032565 (PMC3288111; doi:10.1371/journal.pone.0032565)

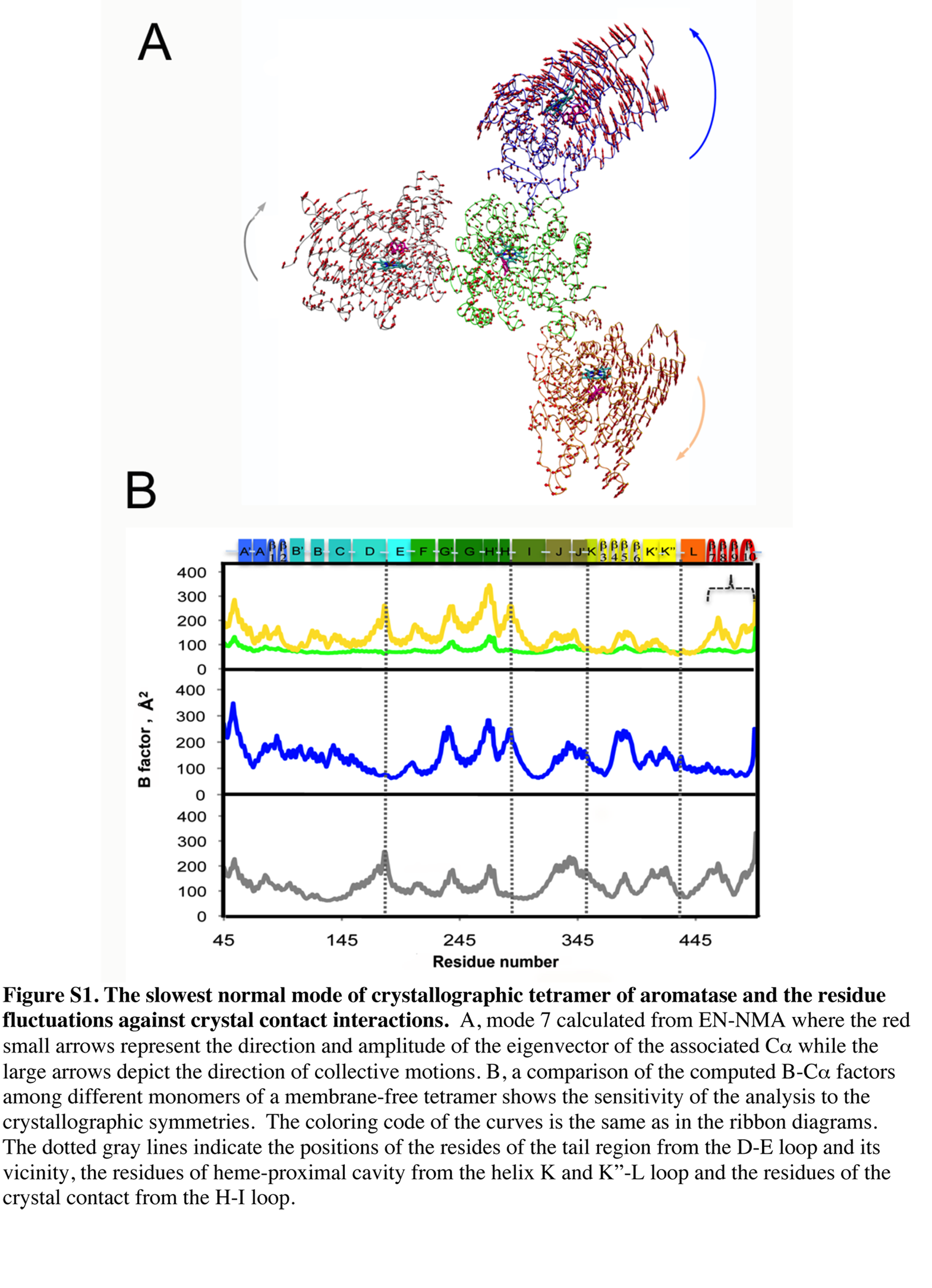

Supplement: Figure S1 — The slowest normal mode of crystallographic tetramer of aromatase and the residue fluctuations against crystal contact interactions. (TIF) [file pone.0032565.s001.tif]

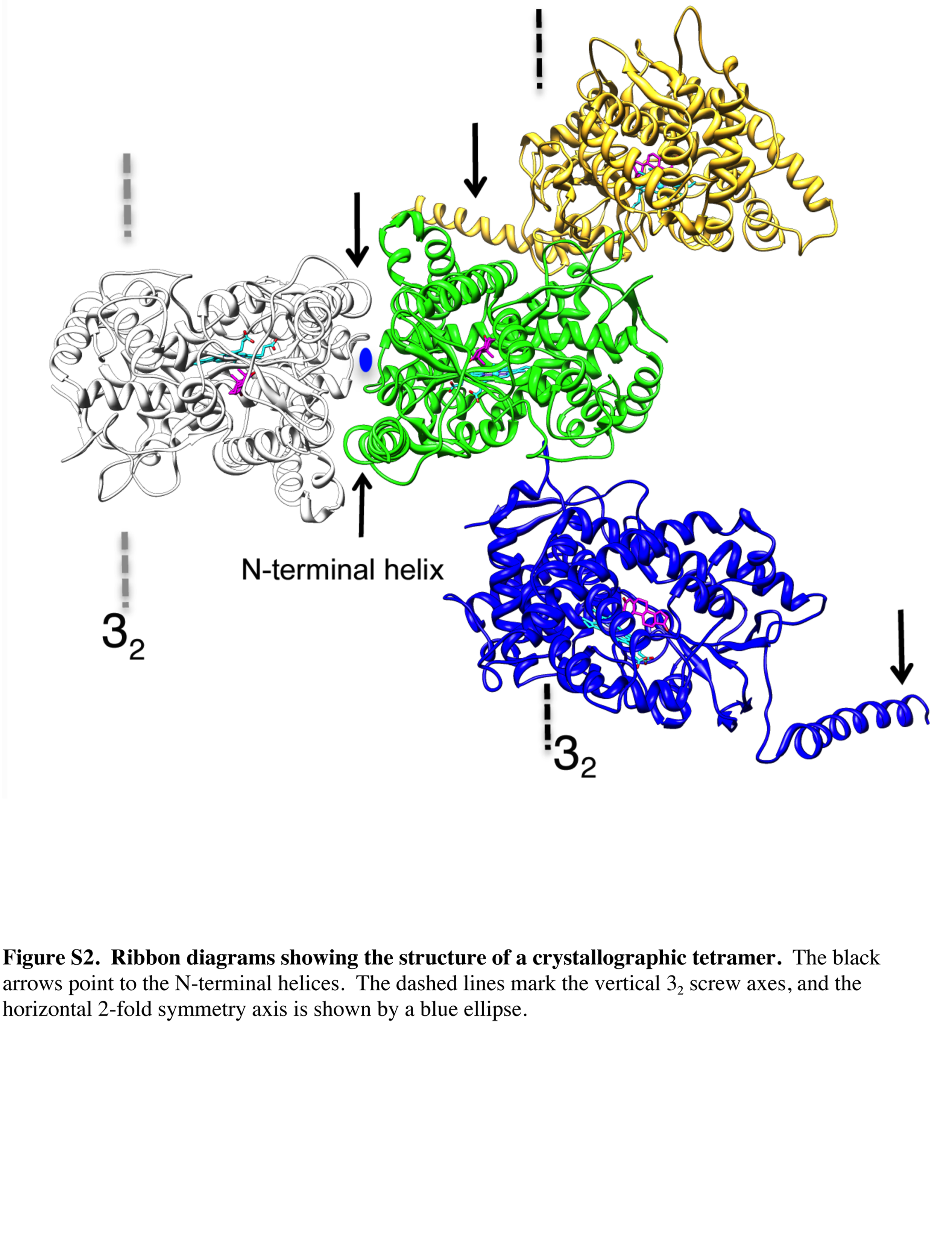

Supplement: Figure S2 — Ribbon diagrams showing the structure of a crystallographic tetramer. (TIF) [file pone.0032565.s002.tif]

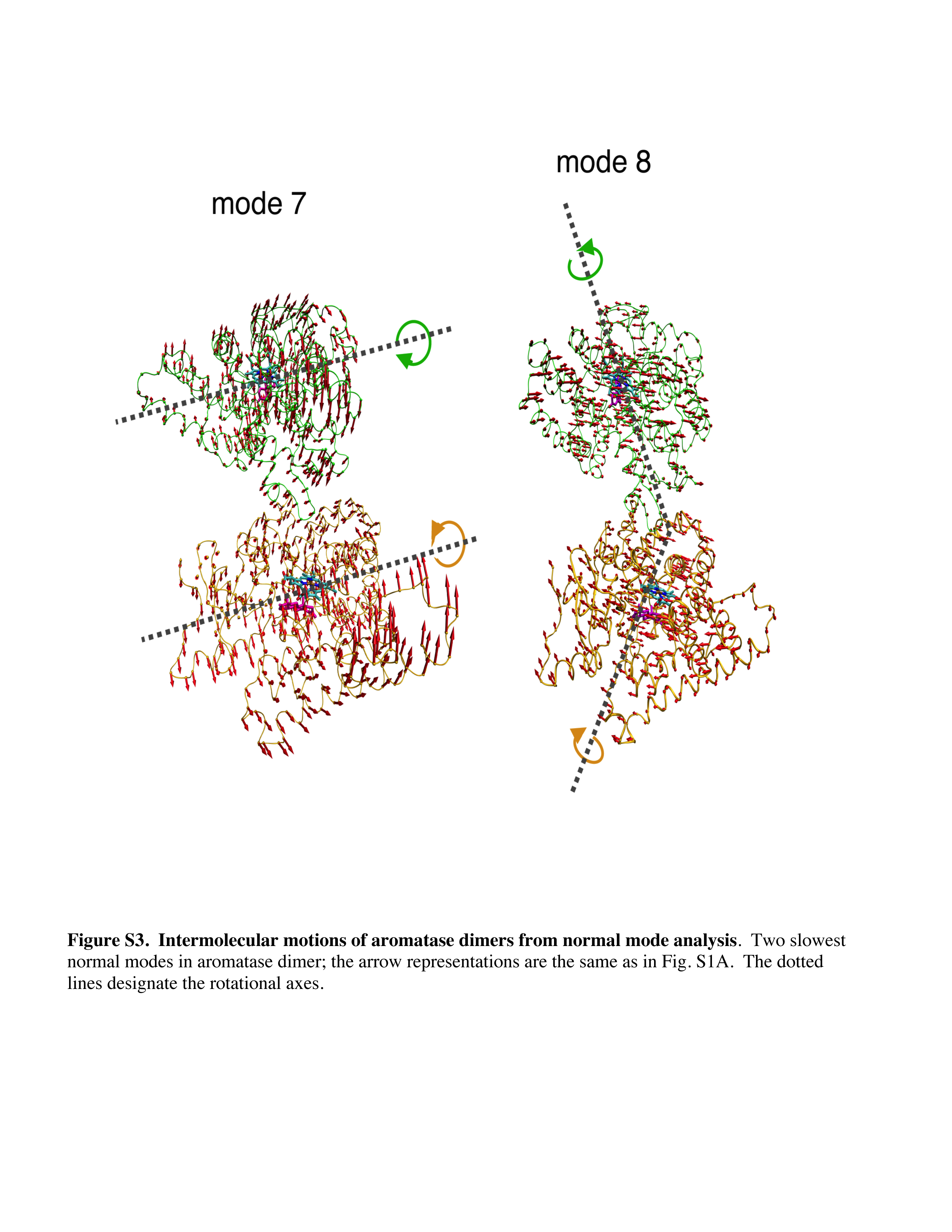

Supplement: Figure S3 — Intermolecular motions of aromatase dimers from normal mode analysis. (TIF) [file pone.0032565.s003.tif]

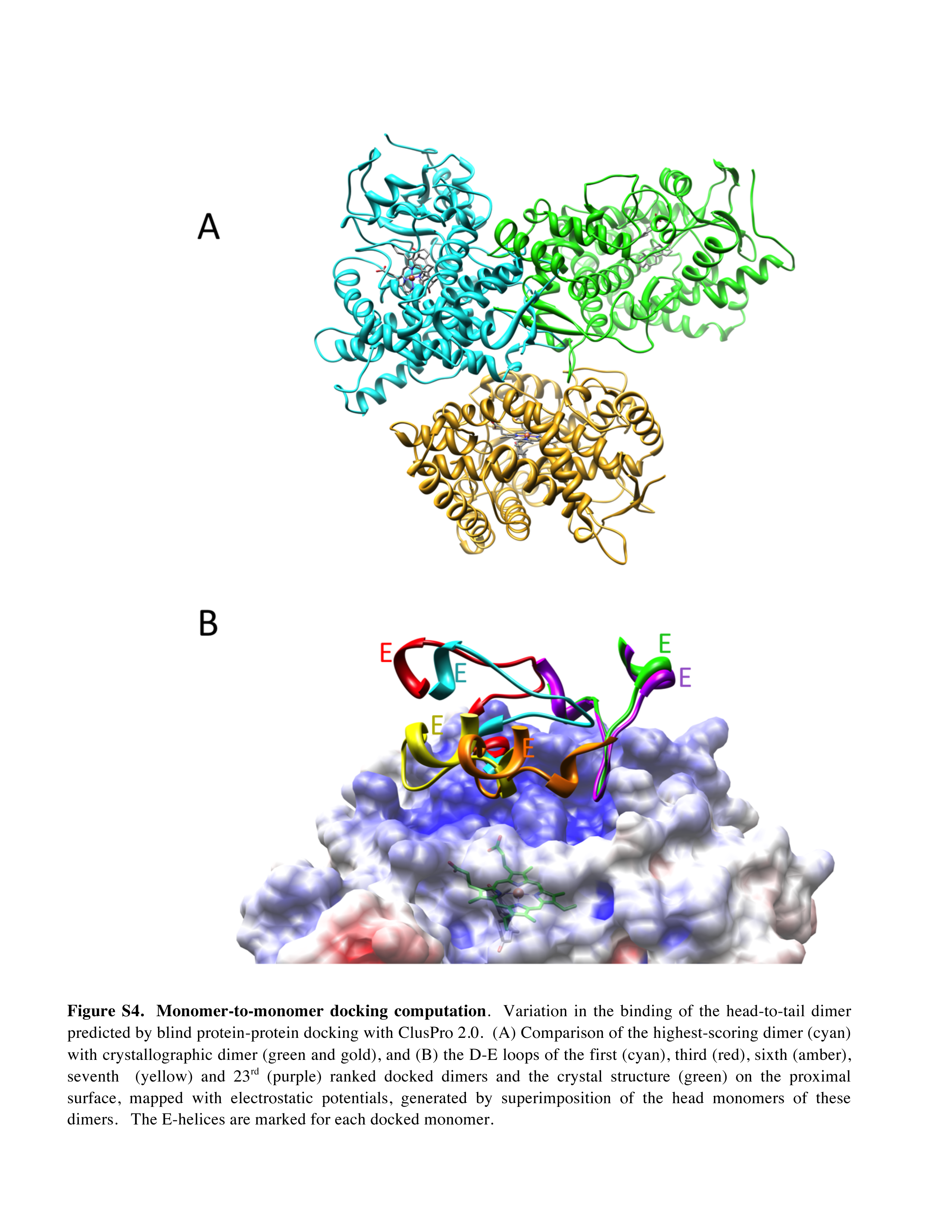

Supplement: Figure S4 — Monomer-to-monomer docking computation. (TIF) [file pone.0032565.s004.tif]

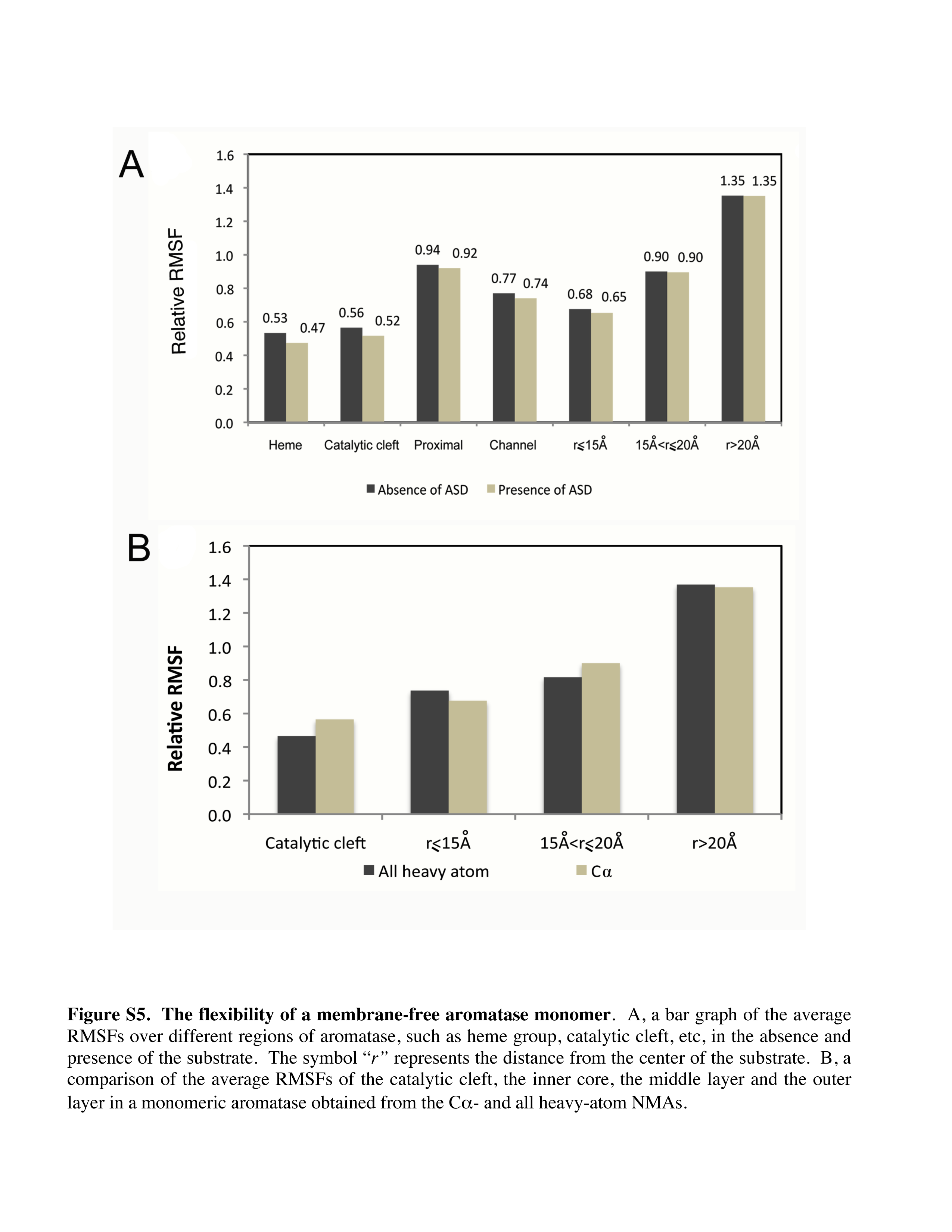

Supplement: Figure S5 — The flexibility of a membrane-free aromatase monomer. (TIF) [file pone.0032565.s005.tif]

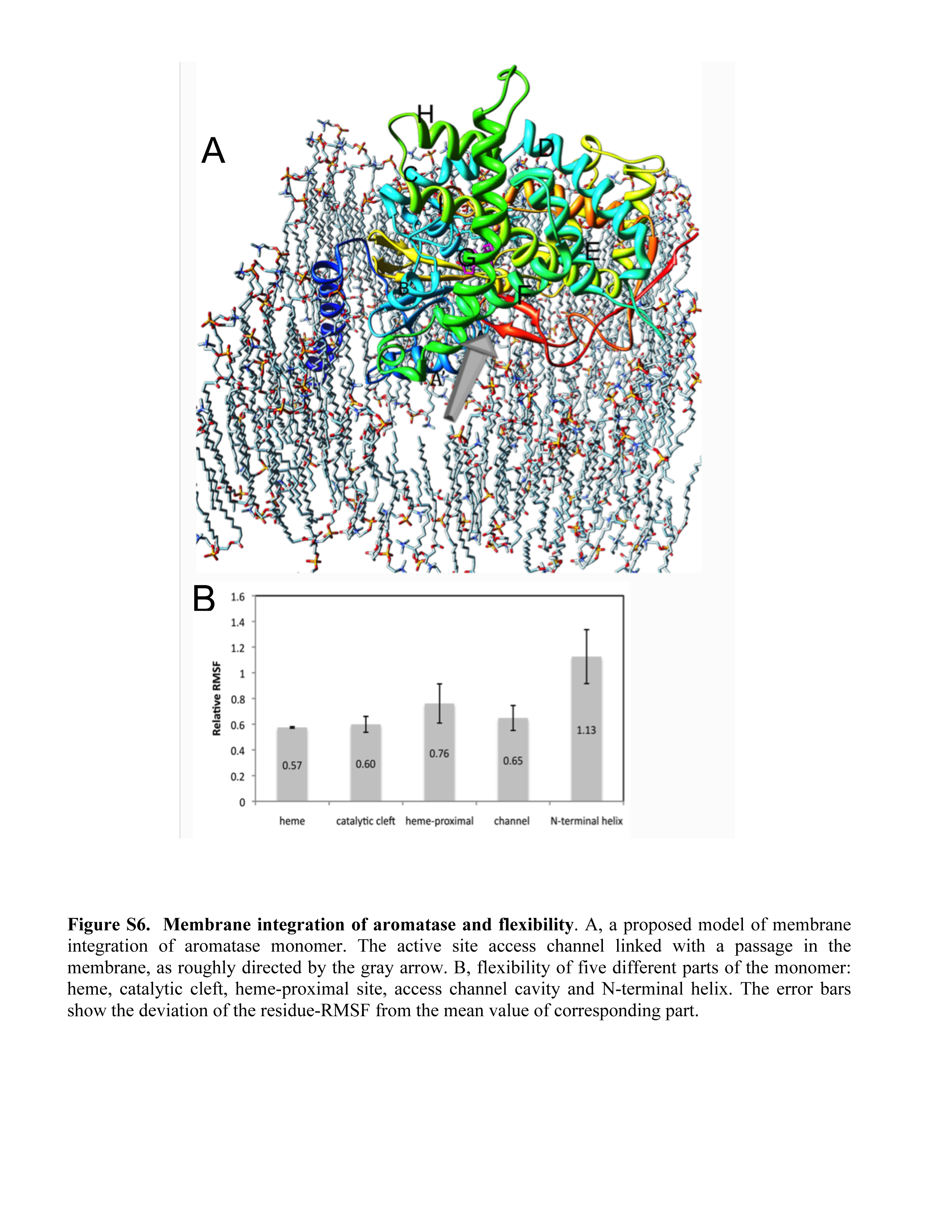

Supplement: Figure S6 — Membrane integration of aromatase and flexibility. (TIF) [file pone.0032565.s006.tif]

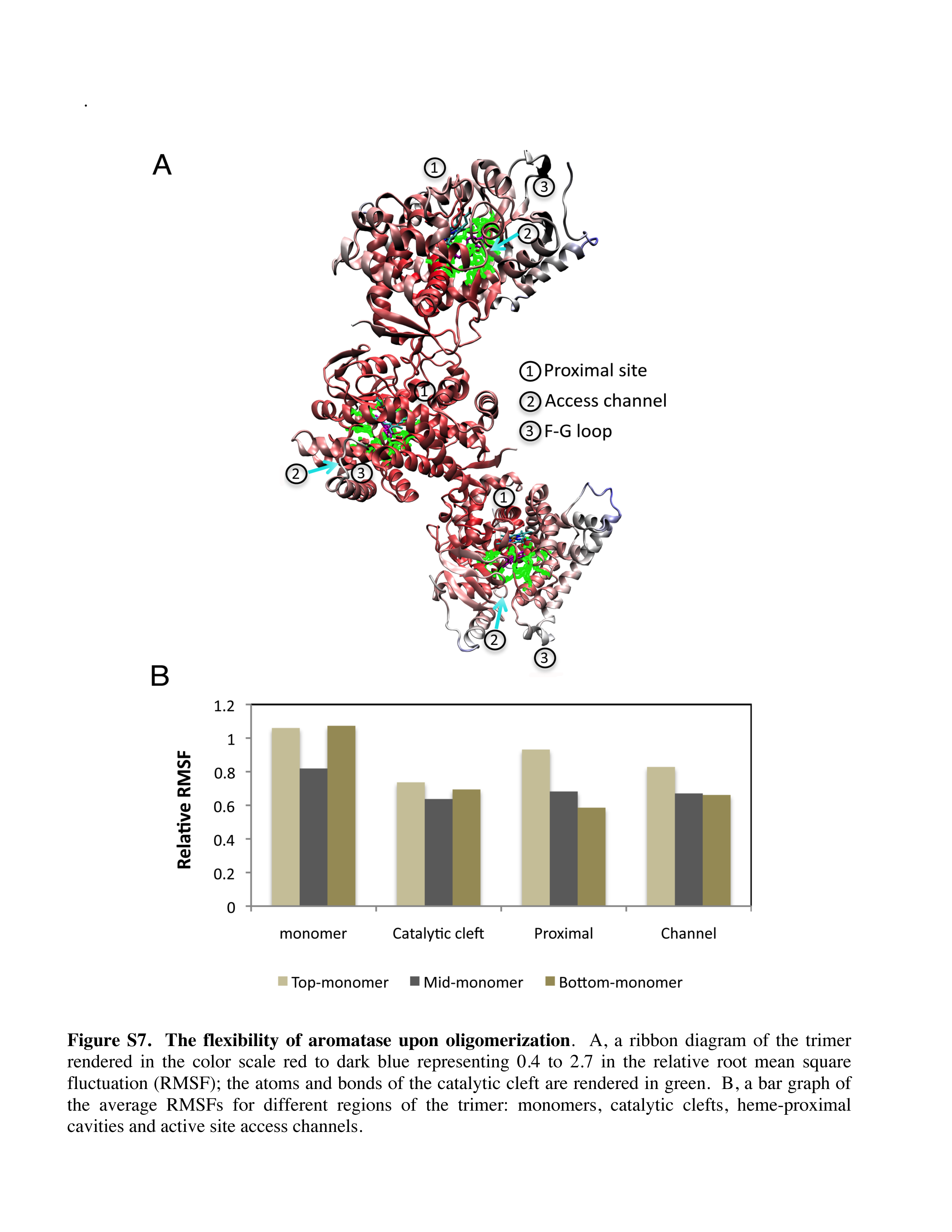

Supplement: Figure S7 — The flexibility of aromatase upon oligomerization. (TIF) [file pone.0032565.s007.tif]

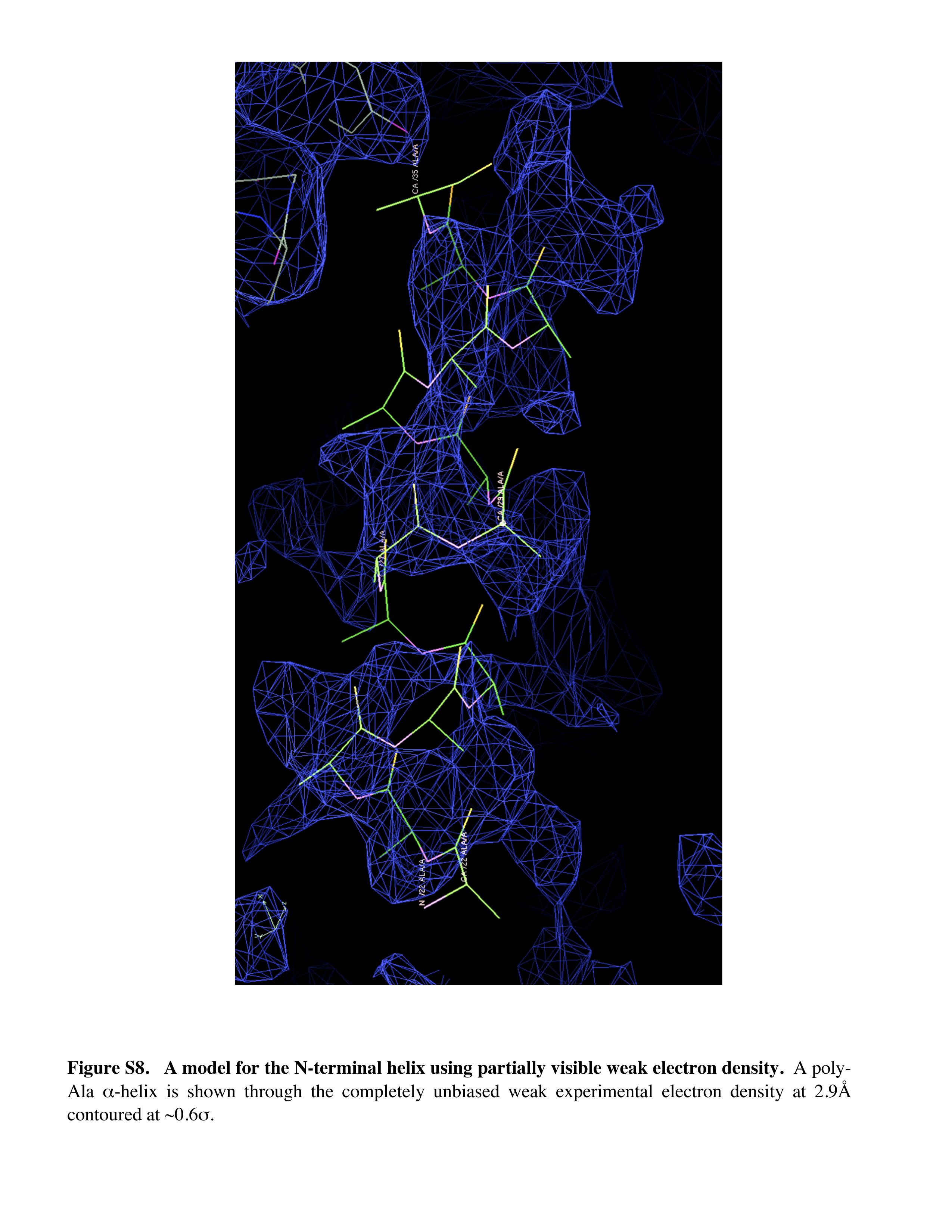

Supplement: Figure S8 — Model for the N-terminal helix. (TIF) [file pone.0032565.s008.tif]
