# Supplementary material for: Motion and Flexibility in Human Cytochrome P450 Aromatase
Source: PLoS One. 2012 Feb 27;7(2):e32565. doi: 10.1371/journal.pone.0032565 (PMC3288111; doi:10.1371/journal.pone.0032565)
Supplement: Text S1 — Probing of other possible oligomeric structures by protein-protein docking computation. (DOCX) [file pone.0032565.s009.docx]

**Text S1, Supporting Information**

**Probing of other possible oligomeric structures by protein-protein docking computation**

A blind aromatase-to-aromatase docking experiment was carried out with ClusPro 2.0 [1]. Top thirty high-scoring models predicted by a scoring function that favors electrostatics and van der Waals interaction were investigated in depth. About two-third of the models predicted a head-to-tail association, similar to the dominant crystallographic interface. Many of the remaining models were judged false positive because the predicted interfaces were at or near the missing 44 amino-terminal residues and had steric violations. Fig. S4A shows a comparison of the top-ranked docking result with the crystallographic dimer; the common “head” monomer is shown in gold. A orientational difference between the two “tail” monomers, described by a rotation of {α,β,γ}={157°,70°,126°} at the proximal site, is also accompanied by a movement of the D-E loop away from the proximal cavity and 8 Å away from that of the crystal structure. Fig. S4B compares the D-E loop locations of the best-docked dimers with that of the crystal structure. The closest model, shown in purple in Fig. S4B, deviates from the crystal structure by 2 Å in backbone RMSD. The D-E loops orient differently across the proximal cavity surface, suggesting flexibility of this mode of intermolecular association. The area buried at the interface for the X-ray structure is 1799 Å^2^. For the docked dimer models it ranges between 1250 and 1800 Å^2^, consistent with the well-known range of 1600±400 Å^2^ for protein-protein recognition [2].

The monomer-to-monomer docking computations were also conducted with GRAMM-X v.1.2.0 [3] using some constraints information from the crystallographic head-to-tail dimer. The highest scoring model was found to be close to the crystal structure with an overall RMSD of 1.8 Å (data not shown), similar to the purple monomer above (Fig. S4B).

**References:**

1. Kozakov D, Brenke R, Comeau SR, Vajda S (2006) PIPER: An FFT-based protein docking program with pairwise potentials. *Prot. Struct. Funct. Bioinfo.* **65**, 392-406.

2. Lo CL, Chothia C, Janin J (1999) The atomic structure of protein-protein recognition sites. *J. Mol. Bio*. **285**, 2177-2198.

3. Tovchigrechko A, Vakser IA (2006) GRAMM-X public webserver for protein-protein docking. *Nucleic Acid Res* 34: w310-w314.
