# Supplementary material for: Motion and Flexibility in Human Cytochrome P450 Aromatase
Source: PLoS One. 2012 Feb 27;7(2):e32565. doi: 10.1371/journal.pone.0032565 (PMC3288111; doi:10.1371/journal.pone.0032565)
Supplement: Text S2 — Fluctuations in an oligomeric aromatase. (DOCX) [file pone.0032565.s010.docx]

**Text S2, Supporting Information**

**Fluctuations in an oligomeric aromatase**

Calculation of the Cα-RMSFs for an aromatase trimer shows that the middle monomer has ~ 20% reduced fluctuation due to self-association. The other two monomers have their overall fluctuation increased by ~7%, more significantly so for the regions farther from the middle monomer (Fig. S6A). The RMSFs of the catalytic clefts, proximal cavities and access channels are systematically compared (Fig. S6B). The computed RMSFs of three catalytic clefts show a deviation of ±5%, roughly one-third of the deviation of their monomers (±15%) indicating that the fluctuations of all catalytic cleft residues are minimally influenced by self association. The proximal cavity, however, has significantly enhanced rigidity when it is involved in the head-to-tail oligomerization for which the reduction in RMSF is 25-35%. The impacts of oligomerization on protein fluctuation at the three channel sites are unequal. The middle and bottom monomers gain in rigidity in their channel sites while the top monomer becomes more flexible.
